# Antenatal depression is associated with perceived stress, family relations, educational and professional status among women in South of China: a multicenter cross-sectional survey

**DOI:** 10.3389/fpsyt.2023.1191152

**Published:** 2023-06-02

**Authors:** Julan Xiao, Ribo Xiong, Yi Wen, Lili Liu, Yueming Peng, Chaoqun Xiao, Caixin Yin, Wenting Liu, Yanling Tao, Fengju Jiang, Min Li, Weixiang Luo, Yu Chen

**Affiliations:** ^1^Department of Thoracic Surgery, Shenzhen People’s Hospital (The Second Clinical Medical College, Jinan University; The First Affiliated Hospital, Southern University of Science and Technology), Shenzhen, Guangdong, China; ^2^Shenzhen Clinical Research Centre for Geriatrics, Shenzhen People’s Hospital, Shenzhen, Guangdong, China; ^3^The Seventh Hospital, Southern Medical University, Foshan, Guangdong, China; ^4^Department of Psychology, School of Public Health, Southern Medical University, Guangzhou, Guangdong, China; ^5^Department of Nursing, Shenzhen People’s Hospital (The Second Clinical Medical College, Jinan University; The First Affiliated Hospital, Southern University of Science and Technology), Shenzhen, Guangdong, China; ^6^Nanfang Hospital of Southern Medical University, Guangzhou, Guangdong, China; ^7^Guangzhou Women and Children’s Medical Center, Guangzhou, Guangdong, China; ^8^School of Nursing, Southern Medical University, Guangzhou, Guangdong, China; ^9^Longgang Central Hospital of Shenzhen, Shenzhen, Guangdong, China; ^10^Boai Affiliated Hospital of Southern Medical University, Zhongshan, Guangdong, China

**Keywords:** antenatal depression, epidemic, risk factors, perceived stress, multicenter cross-sectional study, China

## Abstract

**Background:**

Antenatal depression is a commonly seen mental health concern for women. This study introduced a multicenter cross-sectional survey with a large sample to provide new insights into pregnant women’s depression, its socio-demographic and obstetric characteristics correlates, and its perceived stress among Chinese pregnant women.

**Methods:**

This study conducted an observational survey according to the STROBE checklist. The multicenter cross-sectional survey was performed from August 2020 to January 2021 by distributing paper questionnaires among pregnant women from five tertiary hospitals in South China. The questionnaire included socio-demographic and obstetrics information, the Edinburgh Postnatal Depression Scale, and the 10-item Perceived Stress Scale. For the analyses, the Chi-square test and Multivariate logistic regression were utilized.

**Results:**

Among 2014 pregnant women in their second/third trimester, the prevalence of antenatal depression was 36.3%. 34.4% of pregnant women reported AD in their second trimester of pregnancy, and 36.9% suffered from AD in third trimester of pregnancy. A multivariate logistic regression model indicated that unemployed women, lower levels of education, poor marital relationships, poor parents-in-law relationships, concerns about contracting COVID-19, and higher perceived stress could aggravate antenatal depression among participants (*p*<0.05).

**Conclusion:**

There is a high proportion of antenatal depression among pregnant women in South China, so integrating depression screening into antenatal care services is worthwhile. Maternal and child health care providers need to evaluate pregnancy-related risk factors (perceived stress), socio-demographic factors (educational and professional status), and interpersonal risk factors (marital relations and relationship with Parents-in-law). In future research, the study also emphasized the importance of providing action and practical support to reduce the experience of antenatal depression among disadvantaged sub-groups of pregnant women.

## 1. Introduction

Maternal antenatal depression (AD) is a non-psychotic depressive episode during pregnancy characterized by low mood, lack of concentration, trouble sleeping and eating, and constant irritability and anxiety (1). There is evidence (2) that AD is associated with adverse effects on the mother’s health and fetus/child’s development, including preterm births, preeclampsia, low birth weight in infants, and devastating emotional and behavioral complications like suicide. It is also suggested that AD is a predictor of postpartum depression, with almost half of episodes onset during pregnancy. Their offspring is more likely to have a higher risk of depression during adolescence (3).

Antenatal depression is a global public health problem affecting one in five women worldwide (2). A systematic review (4) reported a mean point prevalence of 15.6% in low and middle-income countries, although only 8% of all these countries had available data. Studies in high-income countries recorded a prevalence of around 10%, including both major and minor depression (5). AD also varies by trimester. A study (6) in the United States reported the incidence was 10% ~ 20%. A longitudinal study in Spanish pregnant women illustrated that the prevalence of probable depression in the first, second, and third trimesters was 23.4, 17.0, and 21.4%, respectively (7). In China, researchers found that of women with symptoms of depression, 7.4% were in their first trimester, 12.8% in their second trimester, and 12.0% in their third trimester (8). A recent systematic review and meta-analysis (8) revealed that the prevalence of antenatal depression was 19.7%, which was the first meta-analysis of perinatal depression in Mainland China. There is evidence that the prevalence of depressive symptoms during pregnancy could be underestimated in clinical practice, as physical symptoms of pregnancy overlap with the signs of AD (1), and the mental health aspect of pregnancy is a neglected component of care for health-care providers. How the specific context affects AD development and what efforts should be made to identify risk factors to assist in identification, prevention, and treatment continuously attract researcher’s interests.

A pregnant woman’s depression is affected in many ways. In previous studies, stress has proven to be a risk factor for depression (9, 10). It is defined as a disharmony state in the organism or a state of threatened homeostasis or health (11). Allostasis is a psychological stress process that involves the activation of neural and neuroendocrine-immune mechanisms during adaptation to potentially stressful challenges (11). However, the allostasis load can lead to disease over a long time. And Maternal stress is one of the most typical and underappreciated reasons for pregnant women. It leads to increased or decreased glucocorticoids in the fetus, which affects the structural and functional development of the brain (8). Therefore, when maternal face stress or complex events, stress appraisal is critical, influencing their psychosocial outcomes.

The World Health Organization recommends that women attend eight antenatal care visits during pregnancy at a health facility (12). Decreased gynecological and obstetrician visits are associated with AD (13). However, much attention has been paid to eugenics and the physical symptoms of pregnancy in China. It was not until 2020 that the National Health Commission launched a program to include AD screening in regular antenatal care. Guangzhou, Shenzhen, and Zhongshan were the pilot cities where this study was conducted, where the free psychological assessment was provided for pregnant women. AD results from a sophisticated interplay of social, psychological, and biological factors. Discerning AD and its risk factors in clinical settings can inform healthcare providers to adjust antenatal care accordingly. So far, research regarding AD and its predicting factors are limited. While previous research has explained the impacts of stress on depression (9–11), they did not identify their effects on Chinese pregnant women’s depression during the post-pandemic period of COVID-19. They had diversified limitations, for instance, the use of unstandardized psychological measures, and included the samples recruited mainly from a single hospital or city (14). In light of this background, the current study, a multicenter (3 cities referred to 5 hospitals) survey in China, aims to understand the prevalence of AD and explore the effects of perceived stress and potential factors influencing AD among women to inform workable intervention strategies in similar post-public health events.

## 2. Methods

### 2.1. Study design and participants

This is a multicenter cross-sectional study carried out from August 2020 to January 2021 in five tertiary hospitals in Guangdong. We recruited women who were pregnant at 13 ~ 40 weeks, (1) aged between 18 and 49; (2) of Chinese nationality; (3) who provided informed consent. Women with a history or family history of psychiatric disorders were excluded, and we excluded women who could not speak or listen.

The sample size was determined using the single population proportion, assuming 31.4% of women affected by AD and the desire to obtain reasonable estimates at a 95% confidence level and 2.5% margin of error (8). The total sample size was 1787 women, accounting for 35% non-response. A total of 2,110 eligible women were invited to participate; 84 women refused to be enrolled, and 12 returned incomplete questionnaires, resulting in 2,014 women. This study included 2,014 pregnant women, allowing detection of significant differences with a power of 0.93 calculated by GPower software.

This study was conducted in Guangdong, which is by far the most populous province in China. Pregnant women were recruited from the obstetrics units in Guangzhou, Shenzhen, and Zhongshan, a total of 5 third-class hospitals. All investigators received unified training on a questionnaire survey and explained to pregnant women the purpose, the principle of confidentiality, and the requirements of filling in the questionnaire when it was distributed to the obstetrics unit. Investigators conducted the face-to-face interview for data collection. The questionnaire consists of three parts.

The ethics committee approved the study of Nanfang Hospital, Southern Medical University, Guangdong, China (Approval number: NFEC-2021-086), and written consent were sought from all eligible participants.

### 2.2. Measurements

#### 2.2.1. Sociodemographics

The part of the questionnaire was based on literature and reviewed by experts in gynecology and epidemiology, covering the following information: (1) socio-demographic factors such as age, occupation, education, monthly income, household registration place, from single-child family; (2) obstetric data, such as parity, pregnancy planning, pregnancy-related diseases; (3) social profiles such as marital relationship, relationship with parents-in-law, concerns about contracting COVID-19.

#### 2.2.2. Edinburgh postnatal depression scale

The Chinese version of Edinburgh Postnatal Depression was used in this study, and a self-reported 10-item scale screens for depressive symptoms over the past 7 days in pregnant women. EPDS total scores range from 0 to 30, with a higher total score indicating eater de Cox et al. (15) reported a score of 12/13 as a clinical cut-off for significant depressive symptoms and a score of 9/10 suggesting minor depression. Australian guidelines (16). recommend a total score of 9 as the cut-off point for mild depression in perinatal depression and a total score of 13 as the cut-off point for major depression and the referral basis. The current study used the scale to assess AD with a threshold of 9. The reliability and validity of the Chinese version have been confirmed, which were comparable to the original scale (17); Cronbach’s alpha in the current study was 0.861.

#### 2.2.3. Ten-item perceived stress scale

The Chinese version of the 10-item Perceived Stress Scale (PSS-10) was based on the theory of psychological stress and was developed in 1983 by Cohen et al. (18), which was used to measure individual stress levels in the past month. The scale has previously shown good reliability and validity (19). The PSS-10 is a 10-item self-report scale. Each item is rated on a 5-point Likert-type scale, from 0 indicating “never” to 4 indicating “very often.” The total score on the PSS-10 ranges from 0 to 40. Higher scores indicate greater levels of perceived stress. The current study’s Cronbach’s alpha for the PSS-10 was 0.754.

### 2.3. Statistical analysis

Data were double put into Epidata 3.0 and then analyzed by SPSS24.0, conducted by and performed under the direct instruction of two biostatisticians. Cross-tabulations with Chi-square tests were used to assess the significance of differences in socio-demographic characteristics, obstetric data, and social profiles between women who had AD and those who did not. Then the independent variables significantly associated (*p* < 0.05) with AD were considered possible contributing factors and entered into a multivariate logistic regression model. Odds ratios (*OR*s) with 95% confidence intervals (95% *CI*) were calculated to measure the strength of association. A *p* value < 0.05 was considered significant in the analysis.

## 3. Results

### 3.1. Demographics

Table 1 summarizes the Socio-demographic features of the participants. More than three-quarters of the participants (85.1%) were aged less than 35 years old. More than half of the participants (61.7%) had a college levels education, followed by those who had senior high school or less (32.6%). A majority (59.6%) of the participants had full-time jobs. 41.9% of the participants reported a monthly household income of 701 ~ 1,300 dollars. 87.1% of participants were not from single-child families. Distribution by place of household registration showed that 65.4% of the participants were urban residents. 26.6% of participants were in the middle of pregnancy, and 73.4% were in the late of pregnancy.

**Table 1:** Socio-demographic features of study participants (*n* = 2014).

Variables	Frequency	Percentage (%)
*Age*		
< 35	1714	85.1
≥ 35	300	14.9
*Level of education*		
Senior high school or less	656	32.6
College/university	1,243	61.7
Graduate or more	115	5.7
*Occupation*		
Jobless	737	36.6
Full-time job	1,201	59.6
Part-time job	76	3.8
*Average household monthly income*		
<$700	390	19.4
$701 ~ 1,300	843	41.9
>$1,300	781	38.8
*From single-child families*		
Yes	259	12.9
No	1755	87.1
*Place of household registration*		
Suburban	697	34.6
Urban	1,317	65.4
*Hospitals*		
Hospital 1	483	24.0
Hospital 2	311	15.4
Hospital 3	779	38.7
Hospital 4	274	13.6
Hospital 5	167	8.3
*Location of hospitals*		
Guangzhou	1,068	53.0
Shenzhen	167	8.3
Zhongshan	779	38.7

Descriptive statistics.

### 3.2. Prevalence of prenatal depression

A prevalence of 36.3% was found among the 2,014 women who participated in the assessment after 730 tested positives for AD using EPDS at a cut-off value of 9. In the middle of pregnancy, 34.4% (184/535) of pregnant women reported having AD. And AD was found in 36.9% (546/1479) of pregnant women in late pregnancy.

### 3.3. Socio-demographic characteristics of women with AD

The association between socio-demographic characteristics and AD is presented in Table 2. Women with senior high school education or less were significantly more likely to report AD (*p* = 0.005). Similarly, unemployed women or women with part-time jobs had a higher risk of developing AD than their counterparts with full-time jobs (*p* < 0.001). Women with an average monthly household income of $701 ~ 1,300 were also more likely to develop AD (*p* = 0.008).

**Table 2:** Socio-demographic characteristics of women with AD (*n* = 2014).

Variables	EPDS < 9 (1284)		EPDS ≥ 9 (730)		*χ* ^2^	*p*
	*n*	%	*n*	%		
Age					1.61	0.205
< 35	1,083	63.2	631	36.8		
≥ 35	201	67.0	99	33.0		
Pregnancy period					0.511	0.475
Mid-pregnancy	351	65.6	184	34.4		
Late-pregnancy	933	63.1	546	36.9		
Level of education					10.79	0.005^**^
Senior high school or less	393	59.9	263	40.1		
College/university	805	64.8	438	35.2		
Graduate or more	86	74.8	29	25.2		
Occupation						
Jobless	413	56.0	324	44.0	32.78	<0.001^**^
Full-time job	826	68.8	375	31.2		
Part-time job	45	59.2	31	40.8		
Average household monthly income					11.79	0.003^**^
< $700	237	60.8	153	39.2		
$701 ~ 1,300	513	60.9	330	39.1		
< $1,300	534	68.4	247	31.6		
From single-child families					1.87	0.171
Yes	175	67.6	84	32.4		
No	1,109	63.2	646	36.8		
Place of household registration					1.22	0.268
Suburban	433	62.1	264	37.9		
Urban	851	64.6	466	35.4		
Hospitals					12.22	0.016^*^
Hospital 1	299	61.9	184	38.1		
Hospital 2	198	63.7	113	36.3		
Hospital 3	500	64.2	279	35.8		
Hospital 4	162	59.1	112	40.9		
Hospital 5	125	74.9	42	25.1		
Location of hospitals					10.90	0.004^**^
Guangzhou	659	61.7	409	38.3		
Shenzhen	125	74.9	42	25.1		
Zhongshan	500	64.2	279	35.8		

Descriptive statistics; Chi-square test. ^*^p value < 0.05; ^**^p value < 0.01.

### 3.4. Obstetric characteristics of women with AD

Analysis of obstetric data and probable AD is shown in Table 3. Among them, those who had unplanned pregnancies were more likely to have symptoms of depression than those who were planned pregnancies (*p* < 0.001). Additionally, women with pregnancy-related diseases were significantly more likely to suffer AD than women without such experience (*p* = 0.043).

**Table 3:** Obstetric characteristics of women with AD (*n* = 2014).

Variables	EPDS < 9 (1284)		EPDS ≥ 9 (730)		*χ* ^2^	*p*
	*n*	%	*n*	%		
Parity					0.050	0.826
1	595	63.5	342	36.5		
≥ 2	689	64.0	388	36.0		
Pregnancy planning					13.98	<0.001^**^
Unplanned	396	58.1	285	41.9		
Planned	888	66.6	445	33.4		
Pregnancy-related diseases					4.09	0.043^*^
Yes	62	54.9	51	45.1		
No	1,222	64.3	679	35.7		
Abnormal child-bearing history					1.78	0.182
Yes	191	60.4	125	39.6		
No	1,093	64.4	605	35.6		

Descriptive statistics; Chi-square test. ^*^p value < 0.05; ^**^p value < 0.01.

### 3.5. Social profiles of women with AD

For social profiles, Table 4 showed that poor parents-in-law and poor marital relationships were more significantly associated with AD development than those with satisfying relations (*p* < 0.001). COVID-19 was also significantly associated with AD (*p* < 0.001).

**Table 4:** Social profiles of women with AD (*n*  = 2014).

Variables	EPDS < 9 (1284)		EPDS ≥ 9 (730)		*χ* ^2^	*p*
	*n*	%	*n*	%		
Parents-in-law relationships					15.36	<0.001^**^
Satisfying	1,210	64.9	653	35.1		
Poor	74	49.0	77	51.0		
Marital relationships					38.66	<0.001^**^
Satisfying	1,263	65.0	679	35.0		
Poor	21	29.2	51	70.8		
Concerns about contracting COVID-19					103.80	<0.001^**^
Low	454	51.8	422	48.2		
Middle	677	71.1	275	28.9		
High	153	82.3	33	17.7		

Descriptive statistics; Chi-square test. ^**^p value < 0.01, ^***^p value < 0.001.

### 3.6. Univariate logistic regression analysis of influencing factors of AD In women

Table 5 shows the factors associated with AD among women in the study site. Unemployment women were 1.5 times more likely to develop AD than those with full-time jobs (OR = 1.537, 95% *CI*: 1.217 ~ 1.940). However, those with part-time jobs were not significantly more likely to create AD than those with full-time jobs (*p* = 0.108). Similarly, women who had college/university levels of education were 1.7 times significantly more likely to develop AD than those with graduate or more (OR = 1.748, 95% *CI*: 1.085 ~ 2.816). And those with senior high school or fewer levels of education were at greater risk of AD than those with graduate or more (OR = 1.848, 95% *CI*: 1.095 ~ 3.119). Concerning marital relationships, women who reported poor relationships were 4.2 times significantly more likely to develop AD than women who had satisfying one (OR = 4.270, 95% *CI*: 2.280 ~ 7.995).

**Table 5:** Binary logistic regression analysis of influencing factors of AD in women (*n* = 2014).

Covariates	*B*	S.E	Wald	*p*	OR^†^	95% CI^‡^	
						Lower	Upper
Occupation							
Full-time job	—	—	—	—	—	—	—
Part-time job	0.436	0.267	2.670	0.102	1.546	0.917	2.607
Jobless	0.428	0.119	12.920	<0.001^***^	1.534	1.215	1.938
Level of education							
Graduate or more	—	—	—	—	—	—	—
College/university	0.550	0.245	5.038	0.025^*^	1.733	1.072	2.801
Senior high school or less	0.614	0.270	5.176	0.023^*^	1.848	1.089	3.138
Average household monthly income							
< $700	—	—	—	—	—	—	—
$701 ~ 1,300	0.040	0.142	0.080	0.777	1.041	0.788	1.376
Location of Hospitals							
Zhongshan	—	—	—	—	—	—	—
Shenzhen	−0.272	0.251	1.176	0.278	0.762	0.466	1.245
Guangzhou	−0.070	0.134	0.274	0.600	0.932	0.718	1.211
> $1,300	−0.280	0.158	3.141	0.076	0.756	0.554	1.030
Marital relationships							
Satisfying	—	—	—	—	—	—	—
Poor	1.474	0.324	20.756	<0.001^**^	4.366	2.316	8.231
Parents-in-law relationships							
Satisfying	—	—	—	—	—	—	—
Poor	0.494	0.223	4.895	0.027^*^	1.639	1.058	2.540
Concerns about contracting COVID-19							
Low	—	—	—	—	—	—	—
Middle	0.636	0.244	6.785	0.009^**^	1.889	1.170	3.047
High	1.411	0.250	31.752	<0.001^**^	4.098	2.509	6.694
Pregnancy planning							
Planned	—	—	—	—	—	—	—
Unplanned	0.194	0.109	3.202	0.074	1.214	0.982	1.502
Pregnancy-related diseases							
No	—	—	—	—	—	—	—
Yes	0.217	0.213	1.041	0.308	1.243	0.819	1.886
Perceived stress	0.132	0.012	126.547	<0.001^**^	1.141	1.115	1.167

Binary logistic regression analysis. Hospitals was control variable.

† Odds ratio;

‡ Confidence interval;

* *p* value < 0.05;

** *p* value < 0.01, and ***p value < 0.001.

Regarding parents-in-law relationships, women who responded to poor relationships were 1.5 times significantly more likely to develop AD than women who had satisfying one (*OR* = 1.559, 95% *CI*: 1.018 ~ 2.387). Their concerns about contracting COVID-19 also varied the likelihood of AD. Women who perceived a higher likelihood of contracting COVID-19 during the current outbreak were 4.5 times significantly more likely to develop AD compared to women who perceived their likelihood of contraction was low (*OR* = 4.513, 95% *CI*: 2.881 ~ 7.070). There was a significant positive correlation between perceived stress level and antenatal depression score (*r* = 0.388, *p* < 0.001). In addition, women with more significant perceived stress (*OR =* 1.141, 95% *CI:*1.116 ~ 1.168) were also likely to experience greater AD.

## 4. Discussion

This study provides a multicenter cross-sectional snapshot of the prevalence and relevant predictors of AD in five public health facilities piloting the screening of psychological disorders in Guangdong province, China. To our knowledge, this was one of the first studies concerning the psychological responses of pregnant women during the post-pandemic period of COVID-19 in mainland China. National Health Commission of the People’s Republic of China issued the “Work Plan for Exploring Special Services for Depression Prevention and Control” in September 2020, emphasizing the importance of including depression screening during pregnancy and childbirth in routine maternity checkups and postpartum review procedures, enhancing the early detection and early intervention of maternal psychological problems in this key special population, and proposing higher standards for the prevention and control of depression. Our recent study’s emphasis on pregnant women as a special priority demographic for depression during pregnancy is crucial in the context of creating a healthy China. The prevalence rate of AD was as high as 36.3, 34.4% of pregnant women reported reported AD in their middle trimesters of pregnancy, and 36.9% of pregnant women in late trimesters of pregnancy suffered from AD, suggesting the prevalence in pregnancy depends on the woman’s trimester. International studies (5) have shown that the prevalence of depression in pregnancy can vary from 7 ~ 15% in developed countries and 19 ~ 25% in developing countries. A meta-analysis of 95 studies (8) concluded that the mean prevalence of depression across the antenatal period was 19.7%. Hence, the present figure was higher than the AD prevalence reported in developed and developing countries. The prevalence of AD in this study was also higher than that seen in previous studies in China. For example, in Guangzhou in south China, 28.5% of women had elevated levels of antenatal depressive symptoms (20). A recent systematic reviews and meta-analyses reported the prevalence of AD was 20.7% (21). Previous research (31) in the United States found that the prevalence of depression was 10% ~ 20%, which was lower than in the current study. According to a systematic review and meta-analysis conducted in Mainland China (8), 12.8% of pregnant women with depression were in their second trimester, and 12.0% were in their third trimester. However, when compared to these findings, the prevalence of AD in our study was especially high. Higher AD rates in this study may be explained by two evident differences between other studies and the current one. First, the present data were obtained during the post-pandemic period of COVID-19. Although the virus has been contained and domestically transmitted cases have not been reported in China during the study period, the long-lasting effects of this pandemic were far from over. According to stress theory and perceived risk theory, public health emergencies trigger a series of emotional stress responses containing a higher level of anxiety and other negative emotions (22). These negative emotions may reduce the immune functions of people and destroy the balance of their standard physiological mechanisms. Meanwhile, individuals may overreact to the uncertainties of the disease situation. These include how people will abide by social distancing with international cases climbing, the degree of protection provided by the previous infection, and how well vaccines guard against the virus. Therefore, these COVID-19-related factors have helped to add cognitive dissonance and insecurity to women’s mental discomfort, which may result in significant psychiatric morbidities. Second, the prevalence of AD may be affected by the instruments used, different cut-off points of screening tools, and different time points of pregnancy at which AD was assessed. The frequently used EPDS is a nonspecific screening tool, and there is an inconsistency in the use of the cut-off score (23). Considering that a cut-off point of 9 was applied in the current study, a greater prevalence than that found in other studies is not surprising (20). The more commonly studies used threshold scores are ≥ 10 or ≥ 12 points (24), but those threshold scores may not be suitable for Chinese participant’s screening (17), and lower thresholds increase test sensitivity (24). Moreover, typical symptoms of pregnancy, which overlap with depressive symptoms in EPDS (25), may explain the relatively high prevalence of AD in our study. Therefore, the current study highlights the demand to actively carry out routine screening, psychological health education, and early intervention services for pregnant women.

The findings of this study showed that women with no job were significantly more likely to develop AD compared to women with a full-time job, which was consistent with the findings of previous studies that occupation was a risk for AD (26). This might be attributed to the economic difficulties associated with unemployment or taking odd jobs. This situation seemed particularly relevant in the context of COVID-19 post-pandemic, especially its adverse economic effects on regions like Guangzhou and Shenzhen, where foreign trade is the source of wealth. From this perspective, poverty resulting from dangerous and challenging economic circumstances may contribute to anxiety and depression for pregnant women regarding growing financial needs with raising a child (8). Our analysis revealed that AD was more prevalent in pregnant women with lower education levels. The relationship between low educational levels and mental disorders has been elucidated in recent studies from a cohort study from southeast China (27) and a systematic review in Mainland China (8). One possible explanation for the observed associations may be that pregnant women with low levels of education were not inclined to acquire pregnancy knowledge actively, had less access and more difficulty fully understanding, have higher excessive worry about pregnancy and delivery safety and maternal and child health, which somehow increase the occurrence of AD to a certain extent. Future studies could include women from different educational backgrounds to understand better the relationship between education levels and AD among pregnant women.

The study also revealed that poor marital relationship significantly impacts reporting AD, which is highly supported by other studies (26–28). A feeling of isolation and loneliness resulting from poor marital relationships plays a significant role in AD development during antenatal (28). In China, the newlywed couple usually set up homes of their own instead of becoming part of the husband’s extended family. In such cases, receiving support from the husband is a sign of approval and a source of confidence and protection against antenatal depression. According to psychosocial stress theory, social and partner support are essential factors in reducing the risk of depression. A good relationship increases a husband’s support for his pregnant wife.

In contrast, a bad relationship can lead to a sense of worthlessness and despair in pregnant women and, in severe cases, may lead to suicide risk or irreversible mental illness (29). A more pronounced effect was observed when poor marital relationship was reported, particularly in intimate partner violence (26). Research revealed that gender-based violence is the most critical predictor of depressive symptoms in women (30). Despite women’s acceptance of violence under certain circumstances by their husbands as usual in traditional Chinese culture, the current study identifies poor marital relationships during pregnancy, though at a relatively lower percentage, as contributing to AD. In the meantime, our study also revealed that poor parents-in-law relationships significantly impacted reporting AD. Under the background of traditional Chinese culture, some women married and lived with their husband’s families; because of the different growth environments, and cultural backgrounds, some families will produce the different of the concept, such as honor, autonomous in-law conflict is typical family conflict, and parents-in-law relationship satisfaction on the low side of women during pregnancy were more likely too nervous, sensitive, frustration in marriage and family relations, easily lead to the occurrence of AD. Therefore, family members should also pay attention to providing psychological and emotional support to pregnant women, improving the relationship between husband and wife, parents-in-law, and family members, creating a good family atmosphere, and improving marital satisfaction.

In addition, we explored the relationship between mental health and concerns about COVID-19 among pregnant women during the post-pandemic. A higher perceived likelihood of contracting COVID-19 was significantly associated with AD. As Guangzhou is one of the central air transportation hubs with more than 130 international flights connecting significant countries in the world, the potential impact of the global COVID-19 outbreak is high. Amid this moment, some people are behaving recklessly and irresponsibly as if COVID-19 no longer exists. Some evidence suggested that public adherence to COVID-19 preventive and control measures during the pandemic was responsible for lower stress, anxiety, and depression (31). Moreover, professional and authoritative information on COVID-19 received by the whole population contributed to the reduced impact of rumors, which may avoid adverse psychological reactions.

Our study suggested that the perceived stress levels of pregnant women were significantly positively correlated with the score of depression during pregnancy, and perceived stress was a risk factor for AD. Prior studies (32) have shown stress to be shared among pregnant women, and that stress was a predictor of depression. One possible explanation for the observed associations was that childcare and the parenting stress experienced by those women might make them more vulnerable to depression. Another explanation was that pregnant women in their second and third trimesters would give birth due to labor analgesia, security concerns, worries about birth outcomes and fetal health, postpartum body recovery, and a higher pressure level. When these pressures are met with inadequate coping mechanisms, stress disorders develop when these negative emotions are displayed, resulting in depression during pregnancy. It is imperative to identify and evaluate women’s stress during pregnancy, guide pregnant women to adopt appropriate coping strategies for stress debugging and management, and reduce stress perception and depression levels.

Our findings will guide maternal care professionals in tackling mental health issues among pregnant women. Since the development of perinatal mental health services in China is still in its infancy, it is urgent and necessary to integrate antenatal psychological screening into routine maternity care. Furthermore, greater emphasis should be placed on identifying high-risk subgroups and setting up a community support system.

### 4.1. Limitations

This study has several limitations. First, a cut-off point of 9 in screening AD using EPDS may over or under-report the prevalence because of its subjective nature in determining any psychological state, and different cut-off points of EPDS may have impact on the prevalence of AD. Second, the study was conducted in five public hospitals where psychological screening for pregnant women was provided free of charge and did not represent the whole scenario. Third, in the case of reporting marital relationships, women might feel stigmatized for revealing, especially in terms of partner violence, because of social desirability bias.

## 5. Conclusion

The prevalence rate of AD was relatively high in antenatal hospital attendees in Guangdong, China. Unemployed, pregnant women, lower levels of education, poor marital and parents-in-law relationship, more serious concerns about contracting COVID-19, and perceived stress are risk factors that can contribute to AD in the second and third trimesters. The study indicates the necessity of integrating antenatal screening for depression and other psychiatric illnesses with maternal care in China. Providing practical support to women during pregnancy, reducing gender-based violence, and developing culturally tailored programs are critical preventative strategies.

## Data availability statement

The original contributions presented in the study are included in the article/Supplementary material, further inquiries can be directed to the corresponding author.

## Ethics statement

The studies involving human participants were reviewed and approved by the Research Ethics Board of Nanfang Hospital, Southern Medical University of Guangdong Province on March 26th, 2021 (Approval number: NFEC-2021-086). The patients/participants provided their written informed consent to participate in this study.

## Author contributions

YC and WLu designed and managed the study, the methodology was developed by JX, YW, LL, and YP. CX, CY, WLi, YT, FJ, and ML were responsible for the questionnaire survey of pregnant women in five hospitals of South of China and data acquisition. JX and LL analyzed the statistics. JX and RX wrote the first draft of the manuscript. All authors contributed to the article and approved the submitted version.

## Funding

This study was funded by National Natural Science Foundation of China (grant/award number: 71874075), Humanities and Social Sciences Planning Fund of Ministry of Education of China (grant/award number: 18YJAZH008), Construction project of Teaching Quality and Teaching Reform Guangdong Province (Teaching team), grant/award number: Letter of Higher Education from Guangdong (2020) 19 and College Innovation and Entrepreneurship (Employment) Education Project in Guangzhou grant/award number: Higher Education in Guangzhou (2019).

## Conflict of interest

The authors declare that the research was conducted in the absence of any commercial or financial relationships that could be construed as a potential conflict of interest.

## Publisher’s note

All claims expressed in this article are solely those of the authors and do not necessarily represent those of their affiliated organizations, or those of the publisher, the editors and the reviewers. Any product that may be evaluated in this article, or claim that may be made by its manufacturer, is not guaranteed or endorsed by the publisher.

## Abbreviations

AD: antenatal depression
COVID-19: 2019 Coronavirus disease
EPDS: Edinburgh postnatal depression scale
WHO: World Health Organization
